# Electrical stimulation of the cuneiform nucleus enhances the effects of rehabilitative training on locomotor recovery after incomplete spinal cord injury

**DOI:** 10.3389/fnins.2024.1352742

**Published:** 2024-03-26

**Authors:** Myriam I. Scheuber, Carolina Guidolin, Suzi Martins, Andrea M. Sartori, Anna-Sophie Hofer, Martin E. Schwab

**Affiliations:** ^1^Institute for Regenerative Medicine, University of Zurich, Schlieren, Switzerland; ^2^ETH Phenomics Center, ETH Zurich, Zurich, Switzerland; ^3^Department of Neurosurgery, University Hospital Zurich, Zurich, Switzerland

**Keywords:** spinal cord injury, rehabilitative training, deep brain stimulation, mesencephalic locomotor region, cuneiform nucleus

## Abstract

Most human spinal cord injuries are anatomically incomplete, leaving some fibers still connecting the brain with the sublesional spinal cord. Spared descending fibers of the brainstem motor control system can be activated by deep brain stimulation (DBS) of the cuneiform nucleus (CnF), a subnucleus of the mesencephalic locomotor region (MLR). The MLR is an evolutionarily highly conserved structure which initiates and controls locomotion in all vertebrates. Acute electrical stimulation experiments in female adult rats with incomplete spinal cord injury conducted in our lab showed that CnF-DBS was able to re-establish a high degree of locomotion five weeks after injury, even in animals with initially very severe functional deficits and white matter lesions up to 80–95%. Here, we analyzed whether CnF-DBS can be used to support medium-intensity locomotor training and long-term recovery in rats with large but incomplete spinal cord injuries. Rats underwent rehabilitative training sessions three times per week in an enriched environment, either with or without CnF-DBS supported hindlimb stepping. After 4 weeks, animals that trained under CnF-DBS showed a higher level of locomotor performance than rats that trained comparable distances under non-stimulated conditions. The MLR does not project to the spinal cord directly; one of its main output targets is the gigantocellular reticular nucleus in the medulla oblongata. Long-term electrical stimulation of spared reticulospinal fibers after incomplete spinal cord injury via the CnF could enhance reticulospinal anatomical rearrangement and in this way lead to persistent improvement of motor function. By analyzing the spared, BDA-labeled giganto-spinal fibers we found that their gray matter arborization density after discontinuation of CnF-DBS enhanced training was lower in the lumbar L2 and L5 spinal cord in stimulated as compared to unstimulated animals, suggesting improved pruning with stimulation-enhanced training. An on-going clinical study in chronic paraplegic patients investigates the effects of CnF-DBS on locomotor capacity.

## Introduction

A variety of different types of electrical neuromodulation approaches of the central nervous system (CNS) exist nowadays in clinical routine, e.g., deep brain stimulation (DBS) for Parkinson’s disease (Malek, 2019) and spinal cord stimulation for chronic pain (Sdrulla et al., 2018; Fontaine, 2021), and at the level of clinical trials. Stimulation methods in general range from noninvasive transcranial direct current stimulation and transcutaneous electrical spinal cord stimulation to more invasive methods such as epidural spinal stimulation or DBS. Electrical stimulation can increase the excitability and activity of neuronal networks and thereby generate tonic and rhythmic patterns of motor activity, or also dampen or block circuit activity (Neudorfer et al., 2021). After spinal cord injury (SCI), electrical stimulation is tested for modulating descending and ascending input to the spinal cord, and the activity of local spinal networks in order to improve rehabilitative training and endogenous functional and anatomical recovery processes (Karamian et al., 2022). The combination of intense rehabilitative training enhanced by electrical neuromodulation is a promising treatment option which could yield a substantial improvement in motor functionality (Arber and Costa, 2018; Hofer and Schwab, 2019).

A spinal cord injury disrupts the connection between the brain and the central pattern generators (CPGs), the executive centers for leg/hindlimb movements in the lumbar spinal cord (Ghosh et al., 2010). Following the injury, spontaneous circuit reorganization processes can occur at all CNS levels above or below the lesion (Bareyre et al., 2004; Filli and Schwab, 2015; Asboth et al., 2018; Anderson et al., 2022). Depending on the lesion size, these plasticity processes contribute to various degrees of spontaneous functional recovery. After very large lesions, the few spared descending fibers projecting below the injury are often insufficient to reactivate and control (James et al., 2011) the anatomically intact lumbar CPGs. These residual fibers can, however, be recruited by neuromodulation to restore some information flow between the brain and the lumbar CPGs and induce leg movements and augment spontaneous recovery. A promising target for neuromodulation of the locomotor systems by deep brain stimulation (DBS) is the cuneiform nucleus (CnF), a subnucleus of the mesencephalic locomotor region (MLR; Chang et al., 2021; Fougère et al., 2021; Hofer et al., 2022). The MLR is an evolutionary conserved locomotor control center in the brainstem which is crucial for the initiation, speed, and cessation of locomotion (Shik et al., 1969; Ryczko and Dubuc, 2013). Electrical stimulation of the MLR by DBS in rats with large spinal cord injuries has been shown to enable stepping-like movements of the paralyzed legs and enhance the quality of locomotion after moderate to severe injury (Bachmann et al., 2013; Hofer et al., 2022). The effects are indirect, presumably by the recruitment of reticulospinal neurons located in the gigantocellular nucleus (Steeves and Jordan, 1984; Korte et al., 1992; Kiehn, 2006; Lemon, 2008). The second subnucleus of the MLR, the pedunculopontine nucleus (PPN), has been studied as a potential DBS-target over the past 17 years to alleviate locomotor symptoms of patients with levodopa-resistant freezing of gait in Parkinson’s disease (Thevathasan et al., 2018). The therapeutic effects of PPN-DBS, however, are very often highly variable (Mazzone et al., 2005; Plaha and Gill, 2005; Golestanirad et al., 2016; Wang et al., 2017), and therefore it is not widely established clinically. The main reason for this variability seems on the one hand to be the difficulty of electrode targeting in this small structure deep in the dorsal midbrain; computational modeling studies showed that already a 1 mm target error can decrease target activation in the pedunculopontine nucleus (Zitella et al., 2013). On the other hand, there is increasing evidence suggesting that not the PPN, but rather the CnF, located dorsally to the PPN, should be considered as the therapeutic target for electrical stimulation in patients with movement disorders (Goetz et al., 2019; Chang et al., 2020; Roussel et al., 2023).

For patients with spinal cord injury, neurorehabilitation is currently the only treatment available to improve motor regeneration (Hofer and Schwab, 2019). Experimentally, the direct activation of spinal locomotor centers by epidural or transcutaneous stimulation combined with intense rehabilitative training can lead to limited improvements in mobility in patients with large but anatomically incomplete injuries (Dietz and Schwab, 2017; Terson de Paleville et al., 2019; Lorach et al., 2023). Stimulation of the CnF would have the advantage that a large part of the physiological pathway of locomotor control can be activated by minimally invasive unilateral stimulation. The combination of rehabilitation with electrical stimulation of the CnF bears a great synergy potential, with first preclinical evidence showing that CnF-DBS enabled training enhances the activity-based rehabilitation and leads to improved long-term locomotor function in both subacute and chronic spinal cord injury in rats (Hofer et al., 2022). A first in-man proof-of-concept trial (^1^DBS-SCI, Identifier: NCT03053791) is currently investigating the potential of CnF-DBS to improve gait in chronic incomplete spinal cord injured patients.

In the present study we assessed the potential of CnF-DBS to improve locomotion in a clinically relevant translational setting in rats with incomplete SCI comparable to ASIA C severity in human patients. We applied suprathreshold CnF-DBS restricted to stimulation intensities which enable voluntary locomotion during rehabilitative training. The physically exhausting training was limited to two cycles on three days per week, allowing sufficient rest between training days. Somatosensory feedback transmitted via the proprioceptive system, which interfaces with the CPG neurons at the lumbar level and is involved in the physiological control of locomotor activity, is essential for locomotor recovery after SCI (Dietz, 2002; Taccola et al., 2018; Takeoka, 2020). Therefore, we chose an enriched environment as rehabilitative training setup presenting different surfaces that require gait adaptation and increased sensory input to the injured hindlimbs. While observing a beneficial effect of rehabilitative training alone, we show that CnF-DBS assisted medium-intensity training further improves locomotion during rehabilitation with effects persisting after discontinuation of training.

## Materials and methods

### Experimental design

#### Animals

A total number of 30 adult female Lewis rats weighing 220–250 g (Janvier, France) was investigated in this study. The sample size was based on the results of previous experiments (Hofer et al., 2022). Animals were group housed under a 12-h light – dark cycle (06:00–18:00 light) with food and water *ad libitum.* Animals were granted seven days of acclimatization to the animal facility before being habituated and trained in all setups prior to baseline testing. All experimental procedures and animal care were conducted in accordance with ethical guidelines, conform to ARRIVE (Percie du Sert et al., 2020) guidelines, and have received ethical approval from the Veterinary Office of the Canton of Zurich, Switzerland (license nr. 140/2016).

#### Experimental design

The therapeutic potential of repeated CnF-DBS enhanced medium-intensity rehabilitative training after incomplete SCI was evaluated in 30 animals. Following habituation, unilateral electrode implantation into the CnF was performed, followed by baseline testing and incomplete SCI at spinal level T10 two weeks post-implantation. Randomized stratified sampling [weight and “Basso, Beattie, Bresnahan (BBB) locomotor score “on post-injury day 14] was performed to allocate the animals to either of two groups undergoing two different rehabilitation paradigms: enriched environment (EE) only group (*n* = 13), undergoing voluntary rehabilitative training three times a week in an enriched environment setup; DBS/EE group (*n* = 16), also undergoing voluntary medium-intensity rehabilitative training in the same enriched environment setup but supported by suprathreshold CnF-DBS. Animals underwent their respective rehabilitation paradigm for a period of four weeks, starting two weeks after injury. A three-week retention period followed the completion of rehabilitative training. Functionality of electrodes was verified in all animals before (dpi-7) and after (dpi8) incomplete spinal cord injury, as well as after completion of the rehabilitative training (dpi42) in animals of the DBS + EE group only. Subsequently, animals were bilaterally injected with the tracer biotin dextran amine (BDA) in the gigantocellular reticular nucleus (NRG) and euthanized by transcardial perfusion three weeks after tracer injection. Behavioral testing and rehabilitative training were performed within the first half of the light phase. One animal died during unilateral electrode implantation and three animals were excluded from further analysis post hoc due to too high levels of gray matter sparing at the lesion site (*n* = 2 of the EE only group, resulting in *n* = 11 for analysis; *n* = 1 from the DBS/EE group, resulting in *n* = 15 for further analysis). Locomotor recovery was assessed with the BBB locomotor score (Basso et al., 1995) as primary readout and kinematic parameters analyzed by the Catwalk XT system (Noldus, version 10.6). Secondary outcome measures were the activity during rehabilitative training expressed as distance moved, and fiber density of anterogradely traced reticulospinal fibers originating in the NRG as well as 5-dydroxytryptamine (5HT)-positive structures in the gray matter of the lumbar spinal cord segments L2 and L5. Except for the analysis of directly visible effects of DBS where blinding was impossible, all records were number-coded prior to analysis and investigators were blinded to all groups until the end of behavioral and anatomical data analysis.

### Surgical procedures

#### Unilateral electrode implantation

**Figure 1:**
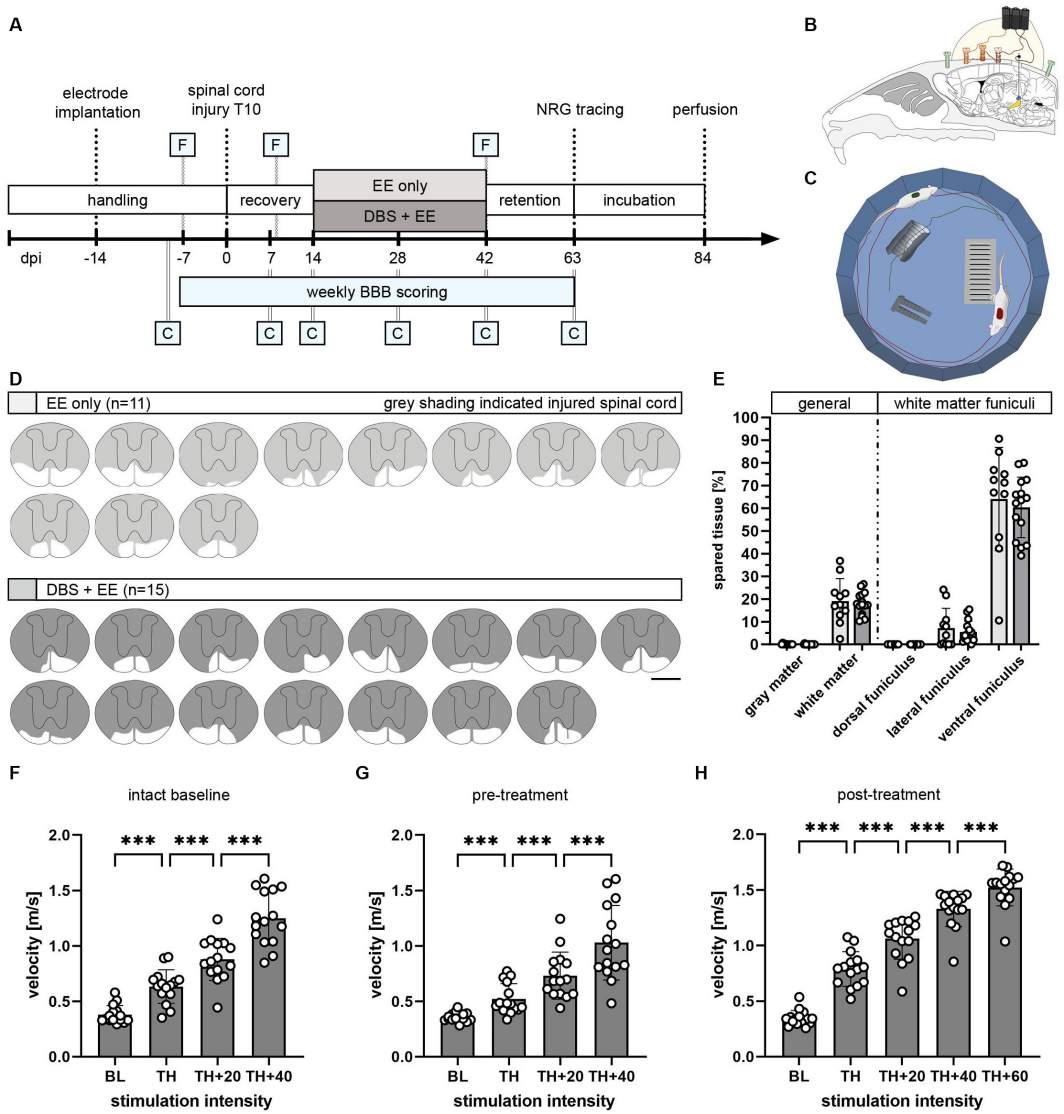
Study design to investigate the therapeutic potential of CnF-DBS after incomplete spinal cord injury. **(A)** Experimental timeline and groups: enriched environment (EE) only (*n* = 11) and deep brain stimulation (DBS) + EE (*n* = 15). F, Testing of functionality of electrodes at selected timepoints. C, Catwalk overground locomotion assessment at selected timepoints. NRG, gigantocellular reticular nucleus. BBB (Basso, Beattie, Bresnahan) score rating of locomotor function in the open field was performed at all indicated time points. **(B)** Schematic illustration of chronic electrode implantation into the left CnF. **(C)** Scheme of enriched environment (EE) setup used for rehabilitative training. **(D)** Lesion size reconstruction of each individual animal. Scale bar: 1 mm. **(E)** Quantified spared tissue of both gray and white matter as well as individual white matter funiculi. One-way ANOVA (*p* > 0.05). **(F–H)** Stimulation intensity dependent increase in locomotor velocity tested at three different timepoints (indicated as F in timeline). Only animals of the DBS + EE group are shown. Repeated measures ANOVA followed by Tukey’s *post-hoc* test. *p < 0.05, **p < 0.01, ***p < 0.001. Data are presented as mean ± SD in **(E,F)**, scatter represents single animals.

Monopolar, 000-gauge stainless steel DBS electrodes isolated with parylene were stereotactically implanted into the left CnF similar to previous publication (Hofer et al., 2022). A 3-pin plug was connected to the electrode shaft and two fine silver-wires, coiled around three screws in the skull plates that served as grounding. Impedance of all electrodes was measured by using an LCR-/ESR-meter (PeakTech^®^ 2,170). Mean impedance of implanted electrodes was 12.29 ± 4.54 kΩ. To enable intraoperative electrical stimulation for optimized CnF targeting, we used 2–5% isoflurane (Piramal Healthcare, anesthesia induction with 5% isoflurane, 2–3% for anesthesia maintenance) followed by intramuscular injection of ketamine (70 mg/kg bodyweight Ketamine, Pfizer). Head-fixation in a stereotactic frame (David Kopf Instruments) allowed precise alignment of bregma and lambda in both the mediolateral (ML) and dorsoventral (DV) plane. After shaving, the surgical area was scrubbed with 70% ethanol and betadine (11 mg/mL, Mundipharma) and protective eye cream (Vitamine A, Braun) was applied. After skin incision, connective tissue was removed before drying and cauterization of the exposed cranium to prevent bleeding and loosening of the implants over time. A circular craniotomy of approximately 4 mm diameter was performed in the left parietal bone using bregma as a reference point. Additionally, five stainless steel screws were inserted into the cranial plate, touching but not penetrating the dura mater: three were positioned in a triangular fashion in the parietal and frontal bone, serving as anchor screws for the grounding of the electrodes, while two screws with a small notch were placed in the elongation of the sagittal suture in the frontal and occipital bone, serving as reference points for later anterograde tracing surgery. Intraoperative stimulation was performed in 21 out of 30 animals to verify optimal electrode positioning. For the intraoperative stimulation, animals were positioned in a hammock ensuring full range of motion of tail and hindlimbs. For the first six animals, initial stimulation was performed at the following coordinates with regard to Bregma: anteroposterior (AP) −7.8 mm, medio-lateral (ML) + 2.0 mm, and in depth from the dura: dorso-ventral (DV) −4.7 mm (AP-7.8/ML + 2 /DV-4.7/0°). The stimulation electrode was then advanced in the DV axis in −0.1 mm steps until observing a replicable rhythmical bilateral hindlimb movement upon stimulation. Confirming our experience from previous experiments^15^, optimal electrode depth was quite consistent between animals, mostly DV-5.3 or occasionally DV-5.2. Proceeding, only these two depths were tested in the following five animals, verifying that DV-5.3 is the predominant optimal implantation depth. Therefore, DV-5.3 was used as implantation depth of the electrode for the subsequent 19 animals, verifying induction of rhythmical bilateral hindlimb movement upon intraoperative stimulation in every second animal (50 Hz, 0.5 ms, 60.93 ± 31.06 μA). Therefore, 9 of these 19 rats were stereotactically implanted without intraoperative stimulation verifying the correct electrode implantation site. Final implantation positions (AP-7.8/ML + 2.0/DV-5.2 to −5.3) were secured with UV-curable dental cement (Tetric EvoFlow, Ivoclar Vivadent). The grounding wires were tightly coiled around the respective screws before the electrode shaft and the grounding screws were fixed with dental cement (Figure 1B). Finally, the skin was sutured and glued with Histoacryl (B. Braun) to the hardened cement cap. Animals were treated with antibiotics (Bactrim, 15 mg/kg bodyweight, Roche) and analgesics (Rimadyl, 2.5 mg/kg bodyweight, Pfizer) for the first seven post-operative days.

#### Spinal cord injury

A cut lesion was used to induce a severe but incomplete SCI which is reproducible and relatively defined in injury size and localization. The spinal cord injury surgery was performed under triple combinational anesthesia containing fentanyl (5 μg/kg bodyweight, Sintetica), midazolam (Dormicum^®^ 2 mg/kg bodyweight, Roche), and medetomidine (Dormitor, 150 μg/kg bodyweight, Provet), in similar technique to previous publication (Hofer et al., 2022). The animals were put in prone position on a warming pad, and a skin incision, separation of the dorsal muscles, and a laminectomy of the T8 vertebra were performed to expose the spinal cord segment T10. After application of additional 10 μL of fentanyl intramuscularly and 1 mL of GlucoSaline solution (3.3% glucose in 0.3% NaCl solution, Braun) subcutaneously, the dura was carefully incised and a subtotal spinal cord transection aiming at 20% bilateral fiber sparing in the ventromedial fiber tracts was performed with iridectomy scissors. After the muscle layers, connective tissue and skin were sutured, animals were allowed an additional 30 min recovery time on a warming pad prior to subcutaneous application of the anesthesia antagonists flumazenil (Anexate, 200 μg/kg bodyweight, Roche) and atipamezole (Antisedan, 750 μg/kg bodyweight, Provet). In the first two weeks following SCI, animals were treated with daily subcutaneous injections of analgesics (Rimadyl, 2.5 mg/kg bodyweight, Provet) and antibiotics (Bactrim, 15 mg/kg bodyweight, Roche). Animals were offered daily high-energy and high-water food and received additional subcutaneous GlucoSaline injections if needed. Micturition was induced manually twice a day throughout the entire study period, and body weight was monitored closely.

#### Anterograde tracing of the gigantocellular reticular nucleus

Unilateral electrical stimulation of the CnF induces a rhythmic bipedal locomotor response, allowing the simultaneous training of both paretic hindlimbs. Therefore, a bilateral anterograde BDA tracing of the main relay station of the MLR, the gigantocellular reticular nucleus, was employed to investigate the stimulation-induced anatomical changes. Animals were deeply anesthetized with fentanyl, midazolam and medetomidine, and put on a warming pad. As the cranial landmarks lambda and bregma were covered by the dental cement cap, the anterior and posterior notched screws were used to retrospectively calculate the coordinates of lambda. Consequently, each individual’s stereotactic frame settings (interaural distance, AP and DV coordinates of the mouthpiece) for head-fixation used and documented during implantation surgery were reapplied and animals head-fixed identically to first stereotactic surgery. Lambda_calc_ was then calculated based on the distance between the original lambda’s coordinates and the reference screws’ coordinates during electrode implantation, and the newly measured AP and ML coordinates from the notches in the tracing surgery. Once the coordinates of lambda_calc_ and the six injection sites (AP −4.6 ± 0.8 mm and ML ± 1.0 mm from lambda_calc_, DV −7.8 mm from dura per coordinate) were retrieved, the posterior screw and posterior part of the cement cap were removed with a drill without damaging the implanted electrode or grounding wires, and a stereotactically guided craniotomy was performed. Per injection site 2 × 120 nL of BDA were injected with an interval time of 3 min between and 5 min after the second injection before retraction of the needle to prevent tracer backflow. A total of twelve stereotactic neuronal tracer injections of 10% biotin dextran amine (BDA, NeuroTrace^™^ BDA-10′000, Invitrogen) were performed per animal using a Nanofil syringe (Hamilton) with a 33 G needle attached to a microinjection pump (UMC4, World Precision Instruments). After the last injection, the craniotomy and the hole in the cement cap was re-filled with dental cement and the skin was sutured and re-glued to the cement cap with Histoacryl. After antagonization of the anesthesia, analgesics and antibiotics were applied daily for one week.

### Behavior assessment and analysis

#### Basso, Beattie, Bresnahan locomotor scoring

BBB scoring (Basso et al., 1995) was used to assess gait quality during open field locomotion in non-stimulated condition. After pre-injury baselines, weekly testing was performed, starting on the 3rd day post-injury. Rats were placed individually in an open field setup and hindlimb motor activity was observed for four minutes. Each session was recorded using an overhead and a lateral camera. Additionally to on-the-spot scoring, two independent researchers (MIS, CG) scored the locomotor performance retrospectively based on the recordings.

#### Gait analysis using the Catwalk XT 10.6

For detailed overground walking performance assessment, we used the CatWalk XT 10.6 (Noldus) system (Hamers et al., 2006). Rats were placed in an enclosed walkway (130 × 68 × 152 cm) which had to be traversed to enter the goal box. Illuminated Footprint™ technology allowed detection and detailed analysis of the animals’ paw patterns recorded with a high-speed video camera positioned underneath the walkway. All animals were habituated and trained in the CatWalk system to cross the walkway without interruptions or hitches. Quality of runs was automatically checked in terms of mean running speed, variation in running speed and maximal run duration. For each animal and timepoint a total of five compliant runs were acquired, and the three most consistent runs were analyzed. In addition to velocity [cm/s], cadence [steps/s], and regularity index [%], stand and swing phase [sec], swing speed [cm/s], step cycle [sec], stride length [cm], body speed [cm/s], mean intensity, base of support [cm], phase dispersions, interlimb coupling, and gait control were analyzed. However, due to the large functional deficit, many of these parameters could not be quantified acutely after injury, as many rats were not able to perform a full step cycle within the length of the enclosed walkway.

#### Verification of correct localization and functionality of the implanted electrode

Additional to the intraoperative stimulation, correct placement and functionality of electrodes was verified at selected timepoints throughout the study period, starting one week post-implantation. In every testing session, each individual’s motor threshold was evaluated, defined as the lowest stimulation intensity (μA) initiating locomotion in stationary animals or eliciting an acceleration of walking speed during ongoing locomotion. Subsequently, the locomotion velocity of 4 consecutive runs was quantified: baseline (BL, without stimulation), at-threshold stimulation (TH), and two supra-threshold stimulation intensities (TH + 20% and TH + 40%). All animals showed an intensity-dependent increase in speed and were thus properly implanted.

#### Rehabilitative training with and without CnF-DBS

Starting two weeks after incomplete SCI, all animals underwent rehabilitative training in an enriched environment setup for four weeks, either with CnF-DBS (DBS/EE, *n* = 16) or without stimulation (EE only, *n* = 13). The enriched environment setups consisted of a round open field setup made of rough synthetic material (80 cm diameter, 30 cm height) containing a small staircase, an adapted horizontal ladder and a creased tunnel (Figure 1C). The rehabilitative training took place three times a week and consisted of two cycles of eight minutes each, separated by 1 h of resting in the home cage. After acclimatization for one minute in the setup, the DBS/EE group received 10 × 10 s of CnF-stimulation to enhance locomotion followed by 30 s of rest after each stimulation during each training cycle, while the EE only group could freely explore the setup for the entire cycle duration. Prior to each training session, bladder management was performed in all animals and individual motor thresholds (TH) of all DBS/EE animals were evaluated at 50 Hz and 0.5 ms pulse width (cathodal pulses, impulse generator and stimulus isolator by World Precision Instruments, for a comparison of different stimulation parameters please see Supplementary Figure 1). Stimulation intensities for the suprathreshold CnF-DBS were limited to a maximum of TH + ≤20% (97.52 ± 84.23 μA; TH + 8.13 ± 7.32%), an intensity range which supports and enables voluntary hindlimb stepping in SCI animals without interfering with animals’ motor control (Hofer et al., 2022) Animals were trained in parallel in pairs of 2 × 2 animals in two identical enriched environment setups, allocated per group (DBS/EE or EE only). Pairing and succession of animals was rotated after each training session, and a pairing was repeated no more than once over the entire rehabilitative training. Each animal’s back was colored for each training, and recording of the training sessions and color coding of the back of the animals allowed an automated quantification of each individual’s covered training distance using EthoVision XT from Noldus (version 11.5).

### Histological analysis

#### Perfusion and tissue preparation

Animals were injected with an intraperitoneal overdose of pentobarbital (300 mg/mL, Streuli Pharma) and transcardially perfused with 200 mL of 1% heparin-Ringer solution (Braun), followed by 300 mL of cooled 4% phosphate-buffered (PB) paraformaldehyde (PFA, pH 7.4, Sigma Aldrich) solution containing 5% sucrose. After dissection, brain and spinal cord were post-fixed in 4% PFA at 4°C for 24 h, before being transferred into 30% sucrose solution at 4°C for at least 3 days for cryoprotection. Following embedding in Tissue-Tek O.C.T. compound (Sakura) and freezing at −40°C for 5 min, coronal sections (14 μm for the MLR, 30 μm for spinal cord and NRG) were collected on slides (Superfrost Plus^™^, Thermo Fisher) or free-floating in 0.1 M PB, and stored at −20°C (free-floating sections in antifreeze solution: 300 g glucose, 1,000 mL 0.05 M PB, and 600 mL ethylene glycol) until further processing.

#### Lesion size assessment

Amount and localization of spared fibers were histologically assessed post-mortem. Serial spinal cord cross sections were stained with Cresyl violet, and lesion sites were reconstructed in a 2D spinal level T10 template based on a spinal cord atlas (Watson et al., 2009) and neuroanatomical studies (Bachmann et al., 2013) using Adobe Illustrator CS6. Percentage of spared fibers was measured at the site of largest lesion extent using Fiji and calculated for white matter (WM), gray matter (GM) as well as the main descending tracts: corticospinal tract (CST), rubrospinal tract (RST), vestibulospinal tract (VST) and reticulospinal tract (ReST). A total of three rats were excluded retrospectively due to too high amounts of spared GM tissue.

#### Quantification of anterograde NRG tracing

The avidin-biotin complex (ABC) technique was used for the detection of the anterograde BDA-NRG tracing in on-slide spinal cord (30 μm, spinal level L2 and L5) and brainstem (30 μm, NRG region) sections. After thawing, sections were washed three times in 0.05 M Tris buffered saline containing 0.3% Triton X-100 (TBS-T) for 30 min each followed by incubation with ABC solution (Vectastain ABC Elite kit) overnight at 4°C in a humidity chamber. Sections were then washed with TBS-T, again three times for 30 min each, and once for 10 min in 0.05 M Tris (pH 8.0) before 10 min pre-incubation in 0.4% ammonium (II) nickel sulfate solution followed by incubation with 3,3′-diabinobenzidine (DAB) substrate and 0.015% H2O2 for 20 min. The reaction was stopped by washing the slides three times for 10 min in cold 0.05 M Tris. After air-drying the sections for 1 h at room temperature and overnight at 4°C, slides were dehydrated through ethanol and xylol (2 × 90%, 2 × 100%, 1 x xylol) before coverslipping with Cytoseal™ 60 (Fisher Scientific). Imaging was performed using Axio Scan.Z1 (Zeiss, 20x) using constant microscope settings across all sections, and the Zen 2 software (Zeiss) was used for Image processing and export in TIFF format. Per animal and lumbar level (L2, L5) three random sections were chosen for analysis. Adobe Illustrator CS6 and Fiji were used for the stereological quantification of NRG-traced fiber density by counting the number of fiber intersections per square on a 10×15 grid superimposed on the gray matter of the spinal cord at a magnification of 40x. While one lumbar spinal cord per group was used for tissue clearing, one animal had to be excluded from the NRG tracing analysis due to weak NRG labeling, therefore, sample sizes for the tracing analysis were *n* = 10 (EE only) and *n* = 13 (DBS + EE) respectively.

#### Immunohistochemistry and analysis

Free-floating spinal cord sections (30 μm, L2 and L5) and on-slide (14 μm, MLR region) brainstem sections were washed 2 × 10 min in phosphate-buffered saline (PBS), followed by 15 min antigen retrieval using immersion in 10 mM sodium citrate buffer at 80°C. After cooling and a 10 min wash in PBS sections were blocked and permeabilized in tris-NaCl blocking buffer (TNB) containing 0.3% Triton X-100 and 5% donkey serum for 1 h at room temperature. After overnight incubation with primary antibodies (rabbit-anti-5HT, 1:2000, ImmunoStar; rabbit-anti-GFAP, 1:1000, Dako; rabbit-anti-Iba1, 1:500, Wako) diluted in TNB containing 0.05% Triton X-100 at 4°C, sections were washed 3 × 10 min in PBS. Sections were then incubated with secondary antibodies (donkey-anti-rabbit-Cy5, 1:500, Jackson ImmunoResearch) for 2 h at room temperature and counterstained with DAPI (1,2000). Following 2 × 10 min washing in PBS and 1× 10 min in 0.05 M Tris, slides were air-dried overnight at 4°C and coverslipped with fluorescence mounting medium (Mowiol, Merck). The fluorescent microscope (Axio Scan.Z1, Zeiss, 20x) was used for image acquisition (exposure times Cy5: 80 ms, DAPI: 7 ms) and the Zen 2 software for image processing and TIFF export. A primary antibody omission control was performed for each marker to assess signal specificity. For 5HT the average axon length in the gray matter of three sections (3 × 30 μm) per lumbar level (L2 and L5) was quantified using AxonTracer (ImageJ). Immune reaction to chronically implanted electrodes was assessed by analyzing the ionized calcium-binding adapter molecule 1 (Iba1) expression, which included counting the absolute number of Iba1+ microglia as well as their morphology (cell body to cell size ratio) on three regions (0.5 × 0.5 mm) around the electrode and the respective regions in the contralateral hemisphere. Perifocal scarring around the chronically implanted electrode was assessed by mean gray value measurement of glial fibrillary acidic protein (GFAP) staining signal across three levels of the electrode channel (from electrode channel to 600 μm into the CNS tissue). All analyses were performed using Fiji. Assessment of perifocal changes upon repeated CnF-DBS was performed in 4 randomly selected animals per group.

#### *In situ* hybridization and analysis

In situ hybridization for neurotensin (Nts) and choline acetyltransferase (ChAT) were used to anatomically verify correct electrode positioning in 14 μm sections of the MLR region in 8 randomly selected animals of the DBS/EE group. The RNAscope Multiplex Fluorescent V2 Assay kit (Advanced Cell Diagnosis) was used following the manufacturer’s instructions for formaldehyde-fixated cryo-sections. Localization of Nts-RNA sequence was used for CnF (Watson et al., 2009; Schroeder et al., 2019) detection (AF647), while labeling of the ChAT-RNA sequence was used to indicate the localization of the PPN (Lein et al., 2007; Schroeder et al., 2019; Ferreira-Pinto et al., 2021; AF555); sections were counterstained with DAPI (AF405). The Axio Scan.Z1 (Zeiss, 10x) and Zen 2 were used for image acquisition and processing (exposure times: AF647 and AF555: 60 ms, AF405: 3 ms). The neurolucida system (MBF Bioscience) was used to produce a 3D reconstruction of Nts + and ChAT+ cells in 14 coronal sections (14 μm, 70 μm intersection distance), covering a range of AP ± 504 μm from the largest extent of the electrode channel. In each section, the localization of all Nts + and ChAT+ cells in the left hemisphere was reconstructed using the widest part of the Sylvian aqueduct as dorsal and medial border for reconstruction, while the medial lemniscus was used as ventral border (Watson et al., 2009).

### Statistical analysis

Data processing, preparation of graphs and statistical analysis was performed in GraphPad Prism 9.5.1. Schematic drawings were created in Inkscape (version 1.0) and figures were generated with Adobe InDesign 2023. Animal groups are consistently color-coded. For analysis, animals were number-coded, and investigators were blinded to groups, conditions, and timepoints until the end of data analysis, except for the acquisition and analysis of the rehabilitative training, where stimulated animals show a visible effect upon CnF-DBS. No statistical outliers were excluded. Bars indicate means ± SD and scatter represent individual animals. Comparison of lesion size of the two groups was done by one-way ANOVA. One-way repeated-measures ANOVA followed by Tukey’s *post-hoc* test to correct for multiple comparison was used to detect differences in locomotor velocity with different stimulation intensities. Šidák’s multiple comparison *post-hoc* test following two-way ANOVA was used to compare stimulation intensities between training cycles. Differences in covered training distance were detected by one-way repeated-measures ANOVA followed by Dunnett’s *post-hoc* test. For BBB score analysis, the BBB score of the better performing hindlimb is depicted for each animal and timepoint. Comparison between different timepoints was performed using two-way repeated-measures ANOVA followed by Tukey’s *post-hoc* test, while comparison between groups was done by Šidák’s multiple comparison *post-hoc* test following one-way ANOVA. One-way ANOVA followed by Šidák’s multiple comparison *post-hoc* test was used to detect differences in the detailed kinematic analysis of overground locomotion. Neuroanatomical spinal cord data was analyzed by Welch’s t-test. *p*-values in text and asterisks in figures indicate significance after *post-hoc* correction for multiple comparison (*p < 0.05, **p < 0.01, ***p < 0.001).

## Results

### Consistent response to unilateral CnF-DBS after incomplete spinal cord injury

The long-term therapeutic potential of repeated CnF-DBS supported rehabilitative training on functional locomotor recovery was assessed by comparing two rehabilitative schemes (Figures 1A–C). Following two weeks of recovery after incomplete thoracic SCI, all animals performed voluntary medium-intensity rehabilitative training three times per week, either accompanied by suprathreshold CnF-DBS (DBS + EE group, *n* = 15) or in the absence of any electrical stimulation (EE only, *n* = 11). Rehabilitative training was performed in a circular environment enriched with obstacles (Figure 1C) to increase sensory input and allow training of gait adaptation in two training cycles of 8 min each per training day. These relatively mild training conditions were chosen to avoid fatigue and mimic a realistic clinical scenario. Quality of locomotor performance was assessed weekly applying the BBB score, while the Catwalk XT system was used at selected timepoints for a more detailed walking analysis (indicated by C in Figure 1A). A three-weeks retention phase was used to assess the persistence of functional improvements. The projections of the gigantocellular reticular nucleus were subsequently traced bilaterally, and animals were perfused after a tracer incubation period of three weeks. Quantification of histological spinal cord lesion site reconstructions showed no significant difference in lesion extent between the two groups with on average < 20% white matter sparing (Figures 1D,E; *p* > 0.05), mainly localized in the ventral funiculus. Accurate targeting and functionality of implanted electrodes were assessed behaviorally and histologically. A gradual increase in stimulation intensity inducing a corresponding increase in locomotion velocity verified proper targeting (Figures 1F–H). Functional targeting was confirmed in all 29 animals before inducing the spinal cord injury (intact baseline) and before treatment group allocation (pre-treatment), while only animals from the DBS + EE group were re-tested before each training during threshold evaluation and after completion of rehabilitative training (post-treatment; indicated with F in Figure 1A). Overall locomotion velocity significantly increased in a stimulation intensity dependent manner (Figure 1F; *p* < 0.001 in Tukey *post-hoc* test after one-way repeated measures ANOVA) in all tested animals at all timepoints.

### Low suprathreshold CnF-DBS supports voluntary medium intensity training resulting in a similar training quantity compared to non-stimulated animals

During rehabilitative training of the DBS/EE group low suprathreshold CnF stimulation of up to TH + 20% was applied, using intensities that initiate or accelerate hindlimb movement but preserve full motor control of animals (Hofer et al., 2022). Stimulation intensities are expressed as a percentage of each individual’s motor threshold (TH), determined at the beginning of each training session. For each training cycle the lowest stimulation intensity between TH and TH + 20% that reproducibly elicited hindlimb movement upon stimulation start was chosen. Over the rehabilitative training period, required stimulation intensities stayed constant with a tendency to slightly but insignificant higher intensities in cycle 2 (*p* > 0.05; except for training day 4, *p* < 0.05; Supplementary Figure 2A). Frequency distribution denoting the relative density of applied stimulation intensities shows that stimulation intensities below TH + 10% were sufficient for initiation of walking in 70.6% of animals in training cycle 1 and in 51.1% of animals in training cycle 2 (Supplementary Figure 2B).

**Figure 2:**
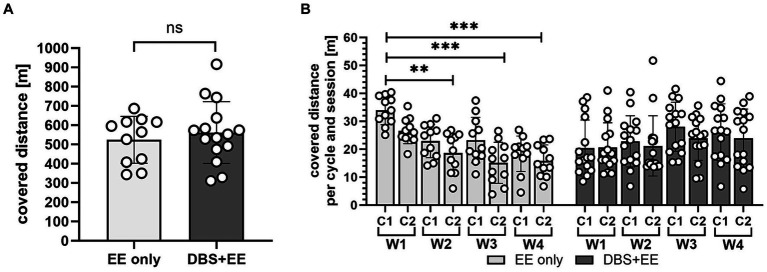
Training quantity did not differ between CnF-stimulated and non-stimulated animals. **(A)** Total covered distance in four weeks of rehabilitative training. EE only, enriched environment only group. DBS + EE, deep brain stimulation and enriched environment group. **(B)** Trained distance partitioned into individual training cycles per week. W, week, C, training cycle. In **(A,B)** asterisks indicate significance of Dunnett’s *post-hoc* test comparison after repeated-measures one-way ANOVA. *p < 0.05, **p < 0.01, ***p < 0.001. Data are represented as mean ± SD, scatter represents individual animals.

While animals from the control group moved continuously during the whole training cycles, stimulated animals tended to rest in the 30 s of rest between the 10 s stimulation intervals. Therefore, due to the overall low suprathreshold stimulation intensity, no significant difference in the total covered distance during locomotor training was found between the two experimental groups (*p* > 0.05). However, in animals that did not receive CnF-DBS the covered training distance significantly decreased over time in comparison to performance during stimulation week 1 (*p* < 0.001, Dunnett’s *post-hoc* test after repeated measures ANOVA; Figures 2A,B). As expected, rats that received a CNF-DBS supported rehabilitative training showed an increased locomotion velocity and improved BBB scores during the stimulation periods (Bachmann et al., 2013; Hofer et al., 2022). However, no obvious changes in the rats’ preferences of using the various facilities throughout the training were observed.

### CnF-DBS supported medium intensity training improves walking quality

**Figure 3:**
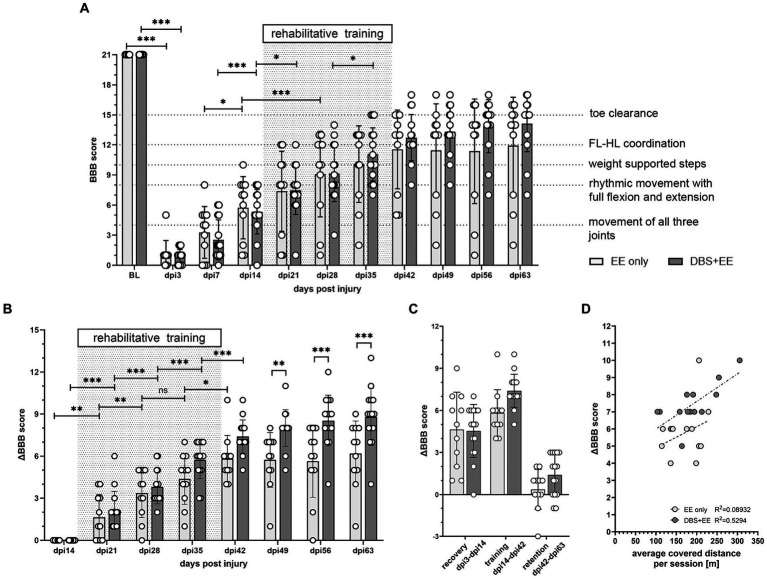
CnF-DBS supported medium-intensity training improves walking quality. Assessment of BBB (Basso, Beattie, Bresnahan) scores was performed without DBS after electrode implantation (baseline, BL), on day post injury (dpi) 3, and weekly from dpi7 to dpi63. **(A)** Recovery of overall BBB scores. Two-way repeated-measures ANOVA followed by Tukey’s *post-hoc* test. FL, forelimb. HL, hindlimb. **(B)** Improvement in BBB score normalized to the locomotor performance level at dpi14, before rehabilitative training start. Asterisks on half-tick line indicate significance of Šidák’s multiple comparison *post-hoc* test comparing the two groups by one-way ANOVA. Asterisks on capped line indicate significant changes within each group between two consecutive timepoints. **(C)** Effect size of ΔBBB improvement during recovery (dpi3-dpi14), training (dpi14-dpi42) and retention (dpi42-dpi63). **(D)** linear regression correlation between overall ΔBBB during training period and average distance moved per training session. *p < 0.05, **p < 0.01, ***p < 0.001. Data represented as mean ± SD. Scatter represents individual animals.

**Figure 4:**
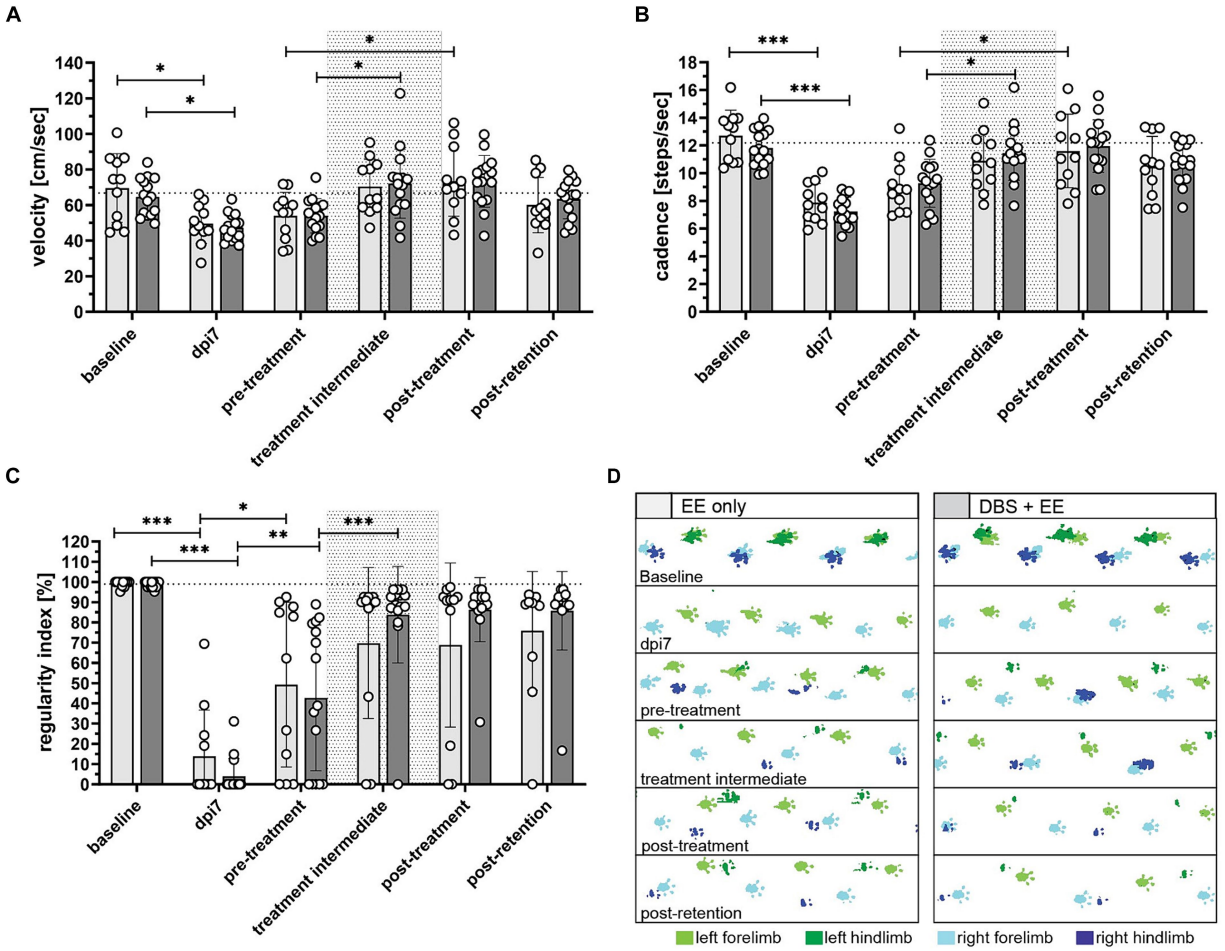
Rehabilitative training improves hindlimb locomotion in CnF-DBS-supported and non-stimulated rats **(A)** locomotion velocity (cm/s), **(B)** cadence (steps/s), and **(C)** regularity index (%) measured using the Illuminated Footprint™ technology of the Catwalk system. **(D)** Representative footprint patterns from catwalk analysis illustrating improvement of hindlimb inter-paw coordination. Green, left paws. Blue, right paws. Light color, forepaws. Dark color = hind paws. Asterisks on half-tick lines in **(A–C)** indicate significance of Šidák’s multiple comparison *post-hoc* test comparing the individual timepoints within each group by one-way ANOVA. *p < 0.05, **p < 0.01, ***p < 0.001. Data are represented as means ± SD, scatter represents individual animals. Dotted area in **(A–C)** indicates rehabilitative training period (dpi14-dpi42). The dotted line in **(A–C)** indicates average intact baseline levels.

Therapeutic effects of CnF-DBS supported training on walking ability were assessed using the BBB score weekly, starting 3 days post injury (Figure 3). Transection of >80% of the lower thoracic spinal cord led to massive impairment of hindlimb function and locomotion as reflected by very low BBB scores 3 days post-injury (Figure 3A). During the first two weeks after injury (recovery phase), animals of both groups exhibited significant spontaneous recovery (dpi14 vs. dpi7; *p* < 0.001 in EE only group, *p* < 0.05 in DBS + EE group; Figure 3A). Non-stimulated animals (EE only) showed significant improvement in overall BBB scores two weeks after rehabilitative training start compared to pre-training level (*p* < 0.001; dpi28 vs. dpi14; Figure 3A). Animals receiving both CnF-DBS and EE exhibited significantly improved hindlimb stepping already within the first week of training (*p* < 0.05; dpi21 vs. dpi14), and also within the third week of training (*p* < 0.05; dpi35 vs. dpi28). After training completion (dpi42), animals receiving electrical stimulation exhibited slightly higher overall BBB scores, resulting in a mean BBB value of 12.7 ± 2.4 compared to 11.6 ± 3.8 in the EE only group. While a functional plateau was observed in non-stimulated animals during the retention phase with a BBB score of 11.9 ± 4.6 at dpi63 (ΔBBB score during retention period of 0.4 ± 1.4), animals from the DBS + EE group showed a slight but insignificant continuation of functional improvement up to a BBB score of 14.1 ± 2.7 at dpi63 (ΔBBB score during retention of 1.4 ± 1.5; Figure 3A). A further improvement resulting from activity and ‘self-training’ in the homecage was exclusively observed in animals with a BBB score ≥13 after completion of the rehabilitative training, while most of the animal with a BBB score ≤10 decreased their locomotor performance during the retention period. Absolute changes in BBB scores (ΔBBB) within a defined experimental period relative to the first ‘base line (BL)’ BBB assessment of this period (for recovery BL BBB: dpi3; for rehabilitative training BL BBB: dpi14, for retention period BL BBB: dpi42) allowed a more detailed investigation of the locomotor recovery and corrected for interanimal variability caused by heterogenous lesion sizes. Analysis of the change of BBB scores (ΔBBB, Figure 3B) over the course of 4-weeks of training shows a significant increase in performance from week to week in both groups (except for a non-significant ΔBBB score in the third training week in the EE only group). Interestingly, the significant decrease in covered distance observed in the control group after the first week of training was not reflected in a lower weekly BBB score improvement in the training weeks 2–4 (Supplementary Figure 3A). After training completion, no significant further hindlimb improvement was observed in both groups. Animals that performed CnF-DBS supported rehabilitative training improved their hindlimb locomotor score to a significantly larger extent than non-stimulated animals at dpi49 (*p* < 0.01), dpi56 (*p* < 0.001), and dpi63 (*p* < 0.001; Figure 3B). While both groups exhibited a similar spontaneous recovery (dpi3-dpi14), CnF-DBS facilitated rehabilitative training resulted in slightly higher levels of BBB score improvements during the training period (dpi14-dpi42) with a beneficial effect persisting beyond the training phase (dpi42-dpi63; Figure 3C; Supplementary Figures 3B–E). Simple linear regression in the correlation between ΔBBB during the training period and the covered distance during training (Figure 3D) shows a significant correlation for the DBS + EE group (*p* = 0.002, Y = 0.01609*X + 4.389, R^2^ = 0.5294) while no significance was observed for the EE only group (*p* = 0.37, Y = 0.01226*X + 3.677, R^2^ = 0.08923). Additionally to the covered distance during the rehabilitative training, percentage of white matter sparing and its localization affects the potential functional recovery in both groups (Supplementary Figures 3B–E). Detailed analysis of overground walking performance at key timepoints (Figure 4) revealed a significant improvement in locomotion velocity after two weeks of rehabilitative training facilitated by CnF-DBS (*p* < 0.05; treatment intermediate vs. pre-treatment), while a similar improvement was found after 4 weeks of training in the non-stimulated group (*p* < 0.05; post-treatment vs. pre-treatment; Figure 4A). Similar improvements were found in the number of steps per second (cadence; Figure 4B). The regularity index, a measure of inter-limb coordination, was heavily impaired 7 days post-injury (Figure 4C). Non-stimulated animals only exhibited a significant improvement during early spontaneous recovery. In contrast, animals receiving CnF-DBS showed a significant improvement during spontaneous recovery as well as in the first half of the rehabilitative training period (Figure 4D as schematic illustration).

### The CnF-DBS facilitated training group shows lower reticulospinal fiber density in lumbar level L2 at 84 days post-injury compared to control group

**Figure 5:**
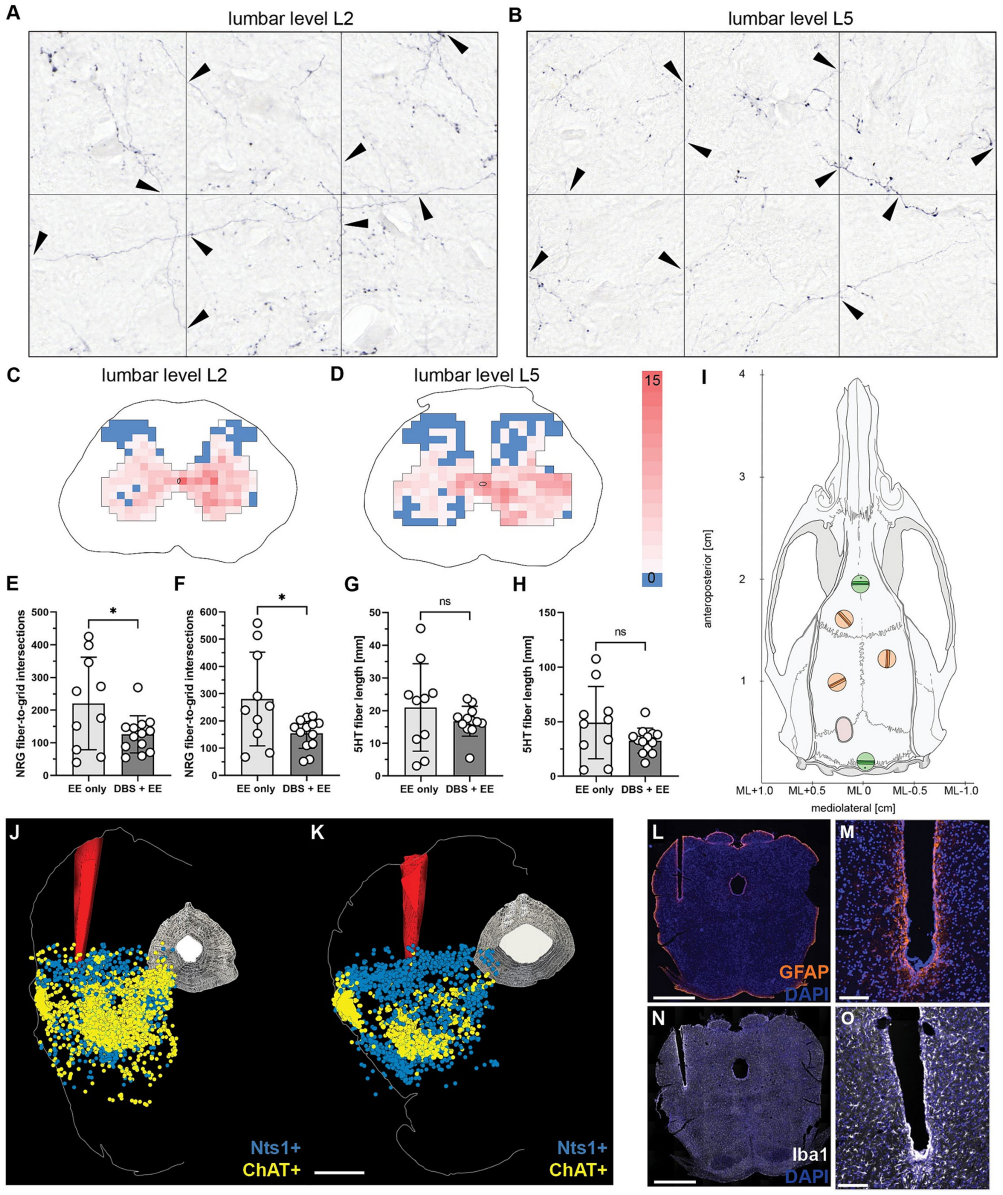
Reticulospinal fiber density in the lumbar spinal cord and reconstruction of electrode implantation site. **(A,B)** Quantification of traced NRG-fiber density at lumbar spinal levels L2 **(A)** and L5 **(B)** by counting fiber-to-grid intersections and creating **(C,D)** fiber density heatmaps of gray matter at spinal levels L2 **(C)** and L5 **(D)**. **(E)** Total quantified NRG-fiber density at L2 and **(F)** L5, respectively. **(G)** Total measured 5HT fiber length in the gray matter at spinal level L2 and **(H)** L5, respectively. **(I)** Reference screws (green) were used to calculate the injection coordinates for the bilateral NRG tracing. **(J,K)** 3D reconstruction of electrode positioning in the cuneiform nucleus of the left mesencephalic locomotor region based on *in-situ* hybridization for neurotensin (Nts, turquoise) and choline acetyltransferase (ChAT, purple) positive neurons of two animals. Scale bar: 1 mm. **(L,M)** Immunohistochemistry for glial fibrillary acidic protein (GFAP) shows minimal perifocal glial scarring around the chronically implanted electrode tip. **(N,O)** Immunohistological staining for ionized calcium-binding adapter molecule 1 (Iba1) demonstrates only minor microglia recruitment and activation around the electrode tip. Scale bar of complete coronal section: 1 mm. Scale bar of magnified electrode tip: 500 μm. **(E–H)** Welch’s t-test. *p < 0.05, **p < 0.01, ***p < 0.001. Data are represented as means ± SD, scatter represents individual animals.

The reticulospinal fibers spared by the large spinal cord lesions were visualized by bilateral BDA tracer injection into the gigantocellular reticular nucleus on dpi63. Animals were euthanized and tissue dissected and fixed on dpi83. Average number of BDA-labeled fibers traversing the lesion site on the level of the largest lesion extent and range of variability were comparable between the groups. Fiber density of BDA-labeled reticulospinal fibers was assessed by stereology (10 × 15 grid intersections) in the gray matter of lumbar segments L2 (Figures 5A,C) and L5 (Figures 5B,D). Reticulospinal fiber density was significantly lower in the CnF-DBS treated group at both levels at this late time point (*p* < 0.05; Welch’s t-test; Figures 5E,F). No significant differences in serotonergic fiber length at lumbar levels L2 (Figure 5G) and L5 (Figure 5H) were found between the two rehabilitative schemes (*p* > 0.05; Welch’s t-test).

### Chronic electrode implantation and repeated CnF-DBS lead to minimal perifocal scarring and inflammation

*In-situ* hybridization for neurotensin (Nts; Lein et al., 2007; Schroeder et al., 2019) was used to localize the CnF and choline acetyltransferase (ChAT; Lein et al., 2007; Jenkinson et al., 2009; Ferreira-Pinto et al., 2021) to localize the PPN in relation to the electrode tip in four randomly selected animals per group. Reconstruction showed that the electrode tip was properly placed within the CnF (Figures 5J,K) in all animals. Perifocal scarring was evaluated by visualization of reactive GFAP-positive astrocytes (Figures 5L,M), while inflammatory changes were assessed by staining for Iba-1 positive microglia (Figures 5N,O). The mean gray value of the GFAP signal within the 250 μm surrounding the electrode channel increased slightly, but insignificantly. Quantification of the absolute number of microglia and their cell body size indicated slight but insignificant immune reaction of perifocal microglia compared to microglia of the contralateral, nonimplanted side. In summary, no significant differences were found between the two experimental groups for both perifocal microglial and astroglial activation.

## Discussion

In this study, we investigated the effects of low- to moderate-intensity rehabilitative training supported by CnF-DBS on functional recovery of locomotor function after anatomically incomplete spinal cord injury in a clinically relevant translational setting. Rats with an incomplete SCI mimicking ASIA C severity in human patients underwent a rehabilitative training program in the subchronic phase after injury, starting two weeks post injury, either without or with support using low suprathreshold CnF stimulation. After completion of the rehabilitative training phase a retention phase ensued in order to assess whether beneficial effects were persistent after discontinuation of training. Afterwards, we bilaterally traced the gigantocellular reticular nucleus in both study groups to investigate potential anatomical rearrangements underlying observed functional effects.

While the mesencephalic locomotor region was previously mainly functionally defined as an evolutionary conserved region able to initiate and modulate locomotion across multiple species (Shik et al., 1969; Dubuc et al., 2008; Ryczko and Dubuc, 2013), latest studies using genetic and viral tools indicate a great diversity of the functions of the MLR (Josset et al., 2018; Ferreira-Pinto et al., 2021; Noga and Whelan, 2022). Converging evidence into a refined model suggests that the pendunculopontine nucleus (PPN) mainly promotes arousal (Stefani et al., 2007; Lee et al., 2014; Roseberry et al., 2016), induces slow exploratory-like locomotor activity via the basal ganglia (Roseberry et al., 2016; Caggiano et al., 2018), or even locomotor arrest (Mitchell et al., 1988; Dautan et al., 2021; Ferreira-Pinto et al., 2021), while the cuneiform nucleus (CnF) seems to consistently orchestrate speed adaptation (Mitchell et al., 1988; Caggiano et al., 2018; Josset et al., 2018) and fast-escape locomotor responses (Goetz et al., 2019). Therefore, the focus is shifting more toward the cuneiform nucleus as the more promising target for stimulation in the treatment of gait deficiencies (Capelli et al., 2017; Chang et al., 2020). We hypothesize that CnF-DBS can activate the remaining, probably mainly reticulospinal, fibers in the ventromedial funiculus after incomplete SCI, and thereby trigger coordinated activity in the sublesional CPGs leading to initiation of stepping and improvement of locomotion (Bachmann et al., 2013; Capelli et al., 2017; Hofer et al., 2022). Our results are in line with the more refined functional description of the two MLR subnuclei, showing a gradual, intensity dependent increase in walking speed at threshold and suprathreshold stimulation of the CnF. Careful determination and re-evaluation of each animal’s motor threshold shows consistent stimulation intensities across the experimental timeline, indicating persistent activation capability of the CnF in the long-run despite repeated stimulation. At higher stimulation intensities (≥ TH + 40%), we observe a characteristic change in locomotor pattern, with a gradual transition from walking to trotting to galloping with left–right synchronization in the hindlimbs, as described previously (Skinner and Garcia-Rill, 1984; Hofer et al., 2022). Restriction to low suprathreshold stimulation intensities (maximal TH + 20%) that trigger locomotion, however, leaving the animals with full context-specific locomotor control is of utmost importance for clinical translation. This stimulation scheme allows locomotor initiation and acute improvement in paretic hindlimb function during CnF-DBS intervals in spinal cord injured rats (Shik et al., 1969; Bachmann et al., 2013; Hofer et al., 2022), supporting but not enforcing locomotion during rehabilitative training. This resulted in a voluntary training paradigm in both the non-stimulated and the CnF-DBS supported rehabilitative scheme, allowing for identification of stimulation vs. training effects.

As voluntary training methods are based on the intrinsic motivation of the rats, we chose an enriched environment as setup for rehabilitative training. Not only do rats housed under standard conditions show a high level of exploration when subjected to an enriched environment (Modlinska et al., 2019), an enriched environment additionally increases the somatosensory feedback that is essential for locomotor recovery after SCI (Dietz, 2002; Taccola et al., 2018; Takeoka, 2020) and challenges the context-specific and coordinated hindlimb function. Spinal cord injured rats from both groups exhibited increased activity in the enriched environment, which resulted in comparable levels of approximately 500 meters of covered distance during rehabilitative training across the four weeks training period. Although the trained distance did not differ between the two groups in any training cycle, interestingly, a significant decrease in the covered distance was found in the non-stimulated control group over the four weeks rehabilitative treatment. The lower extent of trained distance could indicate a decrease in the intrinsic motivation in non-stimulated rats, while CnF-DBS supported animals benefit from the stimulation induced initiation of movement and the improved quality of movement. Furthermore, maintenance of high training distances over time might indicate greater general fitness and endurance of stimulated animals. Importantly, additionally to the quantified trained distance in the enriched environment, rats self-train themselves during daily life while moving about in their home cages (Caudle et al., 2011).

Optimal timing, frequency, and intensity of rehabilitative training to produce a clinically relevant improvement in stepping and locomotor ability are still matter of debate. Most training methods effectively improving over-ground walking speed and distance involve a major component of active walking, with different degrees of assistance. Lower amount of assistance provided can be associated with higher improvements in walking ability (Dobkin et al., 2006; Alexeeva et al., 2011; Field-Fote and Roach, 2011; Lucareli et al., 2011; Yang and Musselman, 2012). While parameters like frequency and duration of rehabilitative training are simple to compare between different rehabilitation schemes, there is no consensus on how to best measure the intensity of training in clinical as well as pre-clinical research (Field-Fote, 2009; Hornby et al., 2011). Clinical rehabilitation training schedules usually comprise 2 to 5 sessions per week with a total of 10 to 130 sessions (Yang and Musselman, 2012), and start in the subacute phase, approximately one month post injury (Chang et al., 2020). Comparison between patients starting rehabilitation early in the subacute phase (<4 weeks) compared to later (>4 weeks) indicated that improvements appear rather during the acute and subchronic phase (Behrman and Harkema, 2000; Dobkin et al., 2006; Yang and Musselman, 2012; Morrison et al., 2018), while a plateau is reached in the chronic period (Kakulas, 2004). Aiming at clinical translation, our rehabilitation paradigm started after two weeks of spontaneous recovery and comprised a total of 12 sessions over a period of four weeks in which rats trained different aspects of hindlimb movement without any assistance, e.g., body weight support. In line with clinical results, conventional rehabilitation alone initiated in the subacute phase leads to improvement in overground walking ability and velocity. CnF-DBS supported rehabilitative training, however, further augmented locomotor recovery on the level of inter-limb coordination and toe clearance. While non-stimulated control animals reached a plateau after discontinuation of the rehabilitative scheme, animals receiving CnF-DBS further improved their locomotor quality even without further electrical stimulation, resulting from further self-training in the homecage. Correlation of the average training quantity with absolute locomotor improvement of paretic hindlimbs shows that despite a comparable covered training distance, rats receiving CnF-DBS improved on average by two BBB scores more than the non-stimulated control. These findings are in line with previous findings, showing an enhanced and persistent gait recovery with CnF-DBS-enabled training in subchronic and chronic spinal cord injury (Hofer et al., 2022). In contrast to the here applied rehabilitative training setup using an enriched environment to stimulate spontaneous activity, the aquatraining used in the earlier study with larger lesions allowed a weight supported training. However, as the non-stimulated control moved significantly lower distances in the weight-supported aqua training setup, the relative contribution of high-intensity training and CnF-DBS to improved locomotor function cannot be fully identified. In our new results, we can show a similar beneficial effect of CnF stimulation on hindlimb recovery compared to our previous study. However, trained distances covered by stimulated and non-stimulated animals were comparable, suggesting that observed functional improvements are rather caused by stimulation than the difference in training intensity (covered distance) and indicating a direct rehabilitative effect of CnF-DBS on gait function.

Due to negligible regenerative capacity, damaged axons of the CNS are unable to re-establish original circuits after injury, resulting in permanent functional impairment (Uyeda and Muramatsu, 2020). The limited spontaneous recovery emerging after incomplete spinal cord injuries is suggested to be the result of plasticity and reorganization of spared descending motor pathways (Maier and Schwab, 2006; Fink and Cafferty, 2016). Supporting this hypothesis, incomplete spinal cord injury induces neuroanatomical plasticity of the corticospinal (Rosenzweig et al., 2010) and the reticulospinal system (Ballermann and Fouad, 2006; Filli et al., 2014) on the level of intra- and supraspinal networks, which are associated with functional recovery. Interestingly, also the MLR exhibits a plastic potential after spinal cord injury, resulting in a 2.5-fold increase of CnF-fibers in the ipsilateral gigantocellular nucleus 35 days after severe incomplete SCI (Hofer et al., 2022). In our study, we investigated the difference in the anatomical rearrangements of sublesional NRG projections between the two treatment groups. The reticulospinal tract originates mainly from the NRG and its fibers travel primarily via the ventral, but also to the lateral, funiculus (Nathan et al., 1996; Lemon, 2008). After large incomplete or even clinically complete spinal cord injuries, ventral tissue bridges are frequently detectable (Pfyffer et al., 2021; Smith et al., 2022), comprising intact remaining parts of the reticulospinal tract (Kakulas, 1999; Taccola et al., 2018; Dimitrijevic and Kakulas, 2020). The spared ventromedial funiculus comprises reticulospinal fibers bridging the incomplete spinal cord lesions in our model, allowing us to trigger coordinated activity in the lumbar CPGs by unilateral electrical stimulation of the CnF. However, the reproducible and relatively defined injury of neuronal pathways in experimentally induced spinal cord injuries differ in some respects from the more complex and often larger lesions seen in human SCI patients.

After completion of rehabilitative training, bilateral anterograde neuroanatomical tracing of the NRG was performed to investigate anatomical rearrangements potentially underlying the enhanced recovery in rats receiving CnF-DBS supported training. Interestingly, the quantification of traced NRG-fibers revealed significantly higher levels of NRG-fiber density in both spinal levels L2 and L5 in the non-stimulated controls compared to the stimulated group. Considering that recovery is suggested to mainly emerge from compensatory sprouting of spared fiber tracts, this finding is counterintuitive at the first glance. However, a closer look at the localization of the traced NRG-fibers hints toward a different potential explanation. In the intact situation, the largest proportion of reticulospinal fibers originating from the NRG terminates in the motor neuron columns of the ventral horn and in lamina 10, with only a small number of fibers terminating in the dorsal horn (Liang et al., 2016). In contrast, in our analysis we consistently found traced NRG-fiber terminations in the dorsal horn across the whole rat cohort, with a slight but insignificant tendency toward higher incidence of traced NRG-fibers in the dorsal horn of the non-stimulated control group (Supplementary Figure 3). This supports the hypothesis that the functional axonal remodeling process after CNS injury recapitulates the pattern of exuberance and pruning seen in early development (Schuldiner and Yaron, 2015). We propose that, while compensatory sprouting of the spared descending motor pathways enables functional recovery of locomotor function, a refined rewiring can be achieved by a training- and use-dependent pruning of exuberant fibers accompanied by stabilization and strengthening of functionally meaningful connections. Evidence from similar functional reorganization processes accompanied by axonal sprouting and pruning has been described following focal binocular lesions in the primary visual cortex of adult macaque (Yamahachi et al., 2009; van Kerkoerle et al., 2018), and after cervical spinal cord injury in rats (Weishaupt et al., 2013). Experimentally, an interesting follow-up experiment investigating this hypothesis would comprise an anatomical time sequence with the analysis of traced NRG-fibers pre- and post-treatment to distinguish between pruning of aberrant fibers vs. lack of sprouting. Furthermore, the CnF-to-NRG connection has been shown to increase more than 2.5-fold five weeks after severe thoracic SCI (Hofer et al., 2022). The analysis of further neuroanatomical changes of CnF projections resulting from CnF-DBS would be interesting but challenging for technical reasons due to the implanted electrode.

Our results show that CnF-DBS supported low- to medium-intensity training further augments the beneficial effect of rehabilitative training on locomotor recovery after incomplete thoracic spinal cord injury in a clinically translational setup. We provide further evidence that stimulation of the CnF enhances both, short-term recovery acutely during the rehabilitative training phase and long-term recovery after discontinuation of stimulation and training. The currently ongoing first in-man proof-of-concept trial (see “Footnote 1”; DBS-SCI, Identifier: NCT03053791) investigating the potential of CnF-DBS to improve gait in chronic incomplete spinal cord injured patients as well as the clinical trial investigating the CnF as therapeutic target to relieve levodopa-resistant freezing of gait in patients suffering from Parkinson’s Disease (see “Footnote 1”; DBS + FOG, Identifier: NCT04218526) are expected to generate further important information on the therapeutic potential of CnF-DBS for gait deficiency in human patients.

## Data availability statement

The raw data supporting the conclusions of this article will be made available by the authors, without undue reservation.

## Ethics statement

The animal study was approved by Veterinary Office of the Canton of Zurich, Switzerland. The study was conducted in accordance with the local legislation and institutional requirements.

## Author contributions

MIS: Conceptualization, Data curation, Formal analysis, Investigation, Methodology, Project administration, Resources, Supervision, Validation, Visualization, Writing – original draft, Writing – review & editing. CG: Data curation, Formal analysis, Investigation, Writing – review & editing. SM: Data curation, Formal analysis, Investigation, Writing – review & editing. AS: Conceptualization, Data curation, Investigation, Project administration, Writing – review & editing. A-SH: Conceptualization, Data curation, Investigation, Project administration, Writing – review & editing. MES: Conceptualization, Funding acquisition, Supervision, Writing – review & editing.

## Footnotes

^1^ https://clinicaltrials.gov
