# Supplementary material for: Electrical stimulation of the cuneiform nucleus enhances the effects of rehabilitative training on locomotor recovery after incomplete spinal cord injury
Source: Front Neurosci. 2024 Mar 26;18:1352742. doi: 10.3389/fnins.2024.1352742 (PMC11002271; doi:10.3389/fnins.2024.1352742)
Supplement: Supplementary file 1 [file Data_Sheet_1.docx]

Supplementary Material

# Supplementary Figures


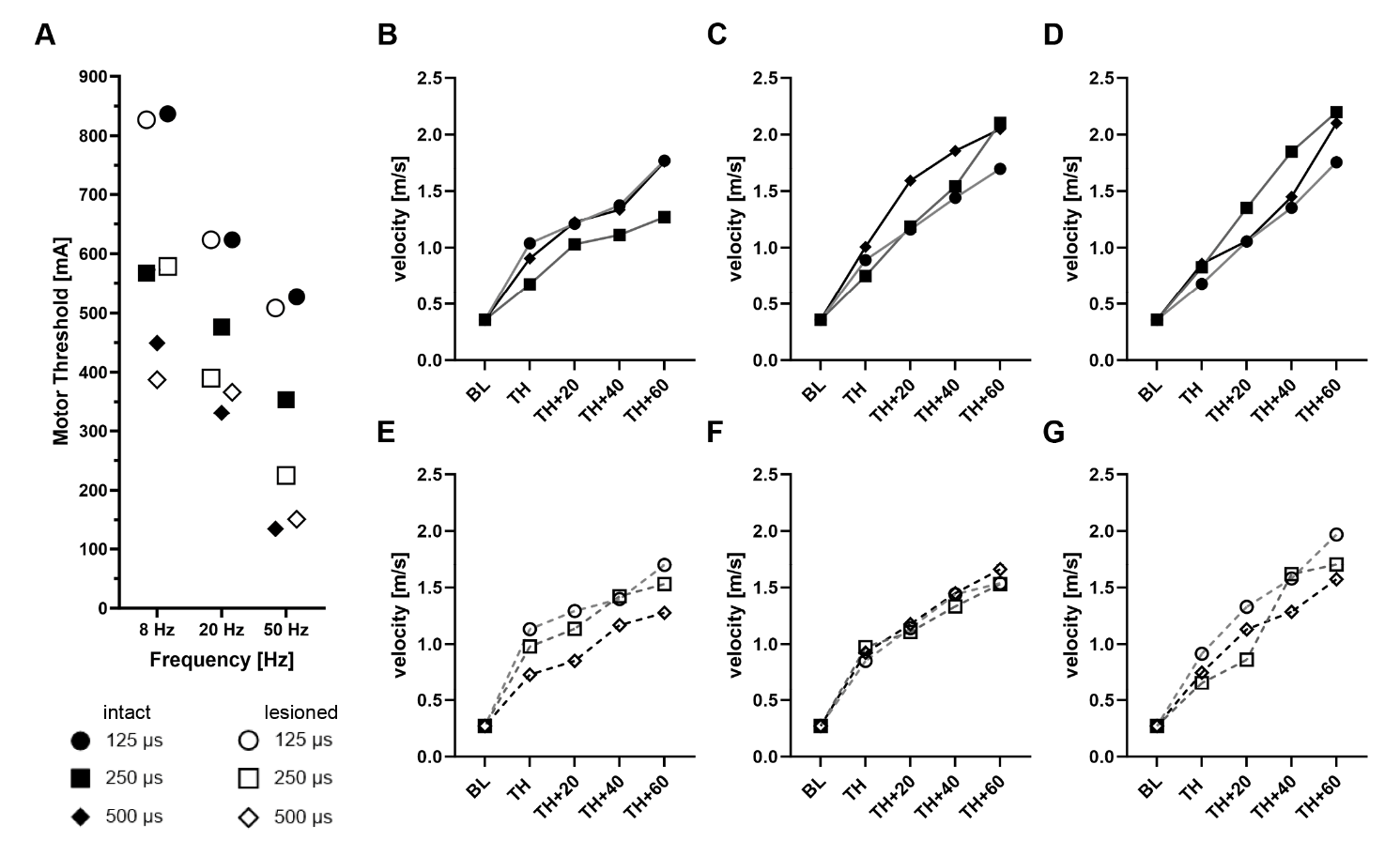


**Supplementary Figure 1. Effect of Different Stimulation Parameter Settings.** Three different frequencies (8, 20, 50 Hz) and pulse widths (125, 250, 500 µs) were tested in n=4 animals in intact and subsequently spinal cord injured condition. (**A**) Average motor thresholds were evaluated with each possible combination of stimulation parameters before and after spinal cord injury. We found that the motor threshold significantly differed between 8, 20, and 50 Hz stimulation for most of the tested pulse widths (p < 0.05); only 125µs pulse width at 20 and 50 Hz, and 500µs pulse width at 8 and 20 Hz showed comparable motor thresholds (*p* > 0.05, repeated-measures ANOVA with Tukey’s post hoc test to correct for multiple comparison). Interestingly, no significant differences were found between intact and spinal cord injured animals. (**B**-**D**) Intensity dependent increase in movement velocity was measured with different pulse width settings at 8 Hz (**B**), 20 Hz (**C**), and 50 Hz (**D**) stimulation frequency in intact and (**E**-**G**) subsequently injured rats at motor threshold intensity (TH) and three suprathreshold intensities (TH + 20%, +40%, +60%) compared to baseline (BL; no stimulation). Data in macaques (86) and humans (26) indicate that PPN stimulation may facilitate or worsen locomotion depending on the electrical stimulation parameter settings. We found no significant differences in the stimulation intensity dependent increase in locomotor velocity, neither between different combinations of stimulation parameters, nor the intact compared to the spinal cord injured condition. To test the beneficial effects of CnF-DBS supported rehabilitative training on locomotor recovery, a stimulation frequency of 50 Hz and a pulse width of 500µs were used.


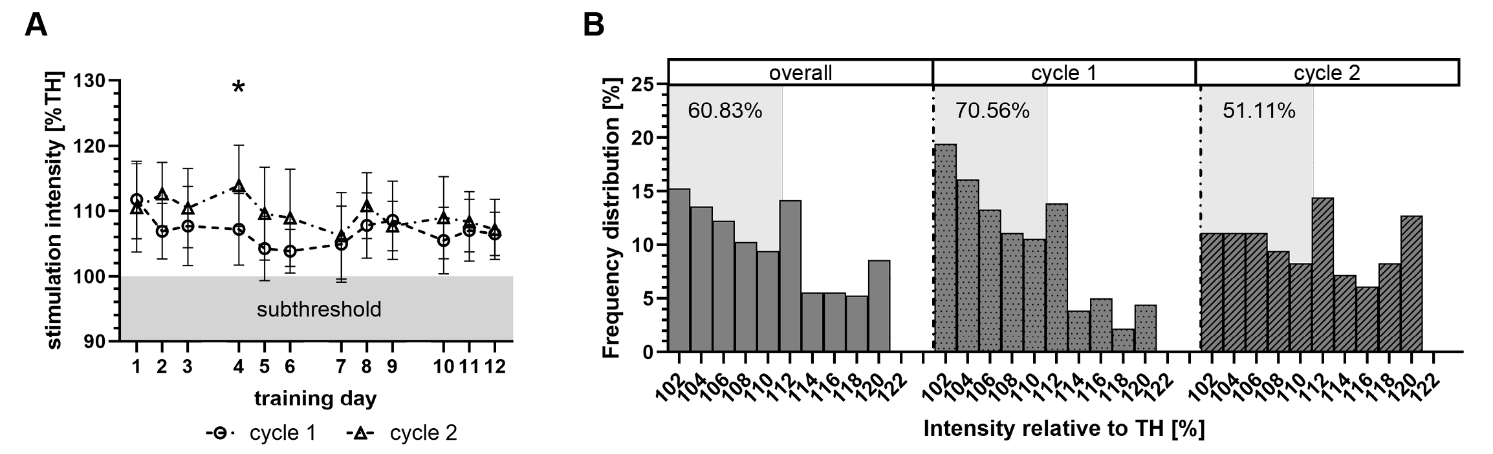


**Supplementary Figure 2. Low- to Medium-Intensity Suprathreshold CnF-DBS was Used to Support Rehabilitative Training.** (**A**) Stimulation intensity expressed as percentage of each individual’s motor threshold (TH) per training cycle and training day. Šidák’s multiple comparison post hoc test following two-way ANOVA. * (*p <* 0.05), ** (*p* < 0.01), *** (*p* <0.001). (**B**) Frequency distribution (%) denoting the relative density of applied stimulation intensities.


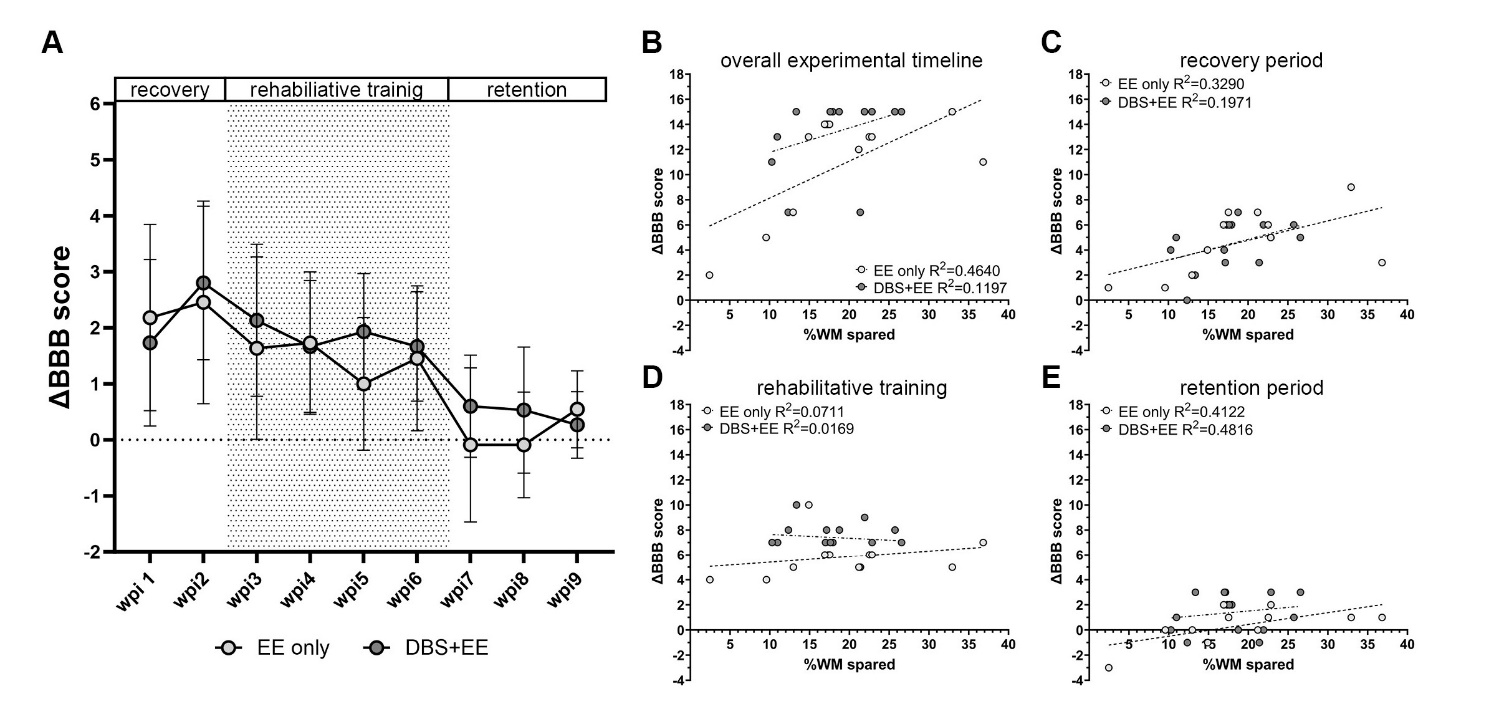
**Supplementary Figure 3. Weekly BBB Improvement and Correlation of the Locomotion Improvement to the Lesion Size.** (**A**) Quantification of ΔBBB scores for each experimental week post SCI shows a slightly higher (*p* > 0.05) weekly ΔBBB in the CnF-DBS group in all experimental timepoints except for the first and the last week. In both groups no significant differences were found within the same experimental phase (recovery: wks1-2; rehabilitative training: wks: 3-6; retention: wks 7-9). However, a significant decrease in ΔBBB score was found between selected weeks from the recovery and training phase when compared to the retention phase in both groups. Šidák’s multiple comparison post hoc test following two-way ANOVA. wpi = weeks post injury. (**B-E**) Simple linear regressions in the correlation between ΔBBB of the different experimental phases and the percentage of white matter (WM) sparing reveals a beneficial effect in CnF-DBS supported animals when compared to non-stimulated control animals with similar lesion sizes. Importantly, this effect is only present during and after CnF-DBS supported rehabilitative training, while no difference between the two groups was found during the two weeks of spontaneous recovery after SCI induction.

|  |
| --- |

**Supplementary Figure 4. Reticulospinal Fiber Localization in the Lumbar Spinal Cord and Reconstruction of Electrode Implantation Site.** (**A-B, E-F**) Fiber density heatmaps of gray matter at spinal levels L2
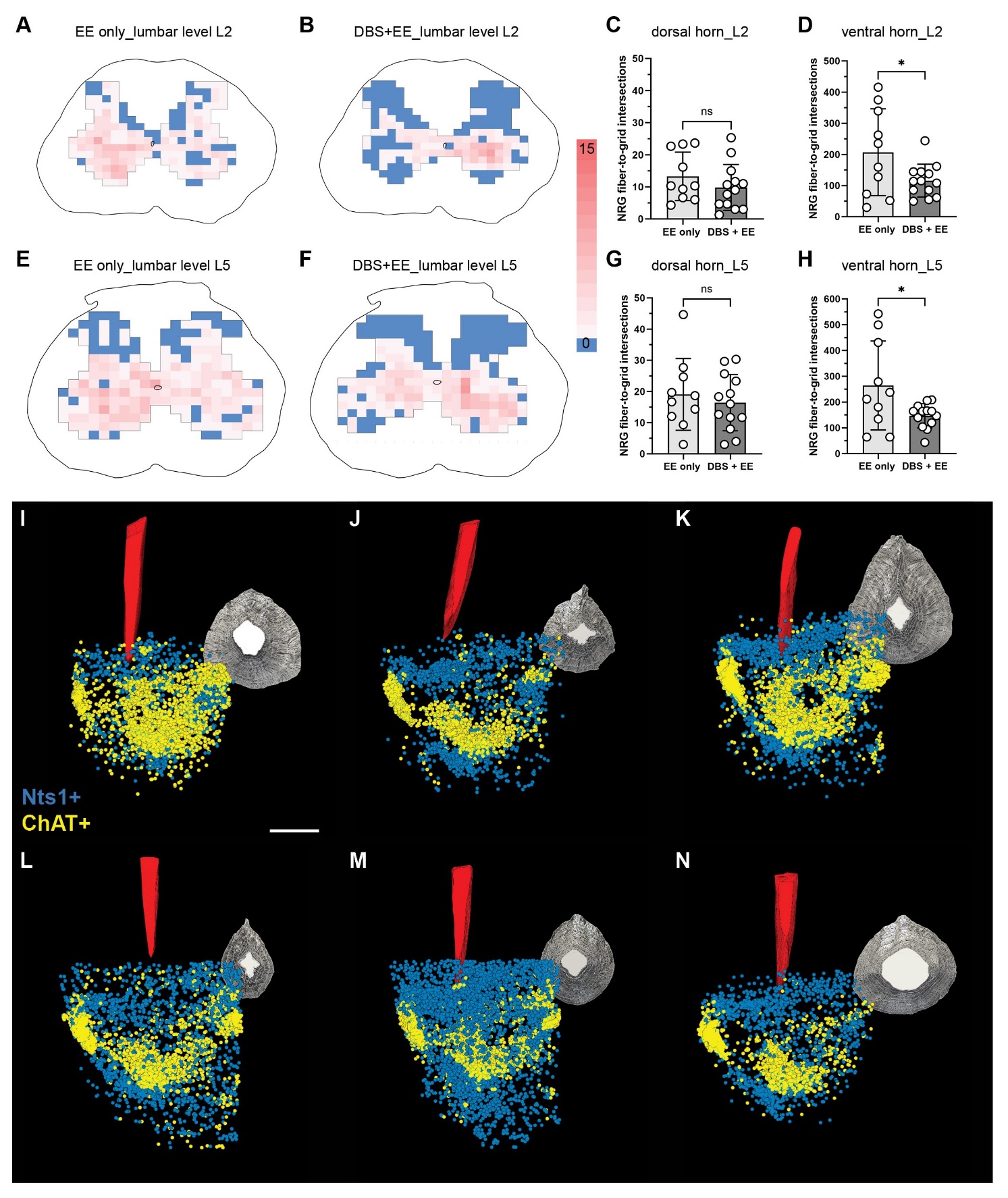
(**A-B**) and L5 (**E-F**) of an animal showing aberrant sprouting in the dorsal horn (**A,E**) and an animal showing only few traced NRG-fibers in the dorsal horn (**B,F**). (**C-D, G-H**) Quantification of traced NRG-fiber density at lumbar spinal levels L2 (**C-D**) and L5 (**G-H**) for the dorsal horn (**C,G**) and the ventral horn (**D,H**). Welch’s t-test. * (*p <* 0.05), ** (*p* < 0.01), *** (*p* <0.001). Data are represented as means ±SD, scatter represents individual animals. (**I-N**) 3D reconstructions of three animals of the control group (**I-K**) and CnF-DBS group (**L-N**) verifying correct electrode localization targeting the CnF (Nts+, blue), rather than the PPN (ChAT+, yellow). Scale bar: 1 mm.
